# Test–retest reliability of a 30-min fixed perceived effort cycling exercise

**DOI:** 10.1007/s00421-022-05094-z

**Published:** 2022-11-27

**Authors:** Callum A. O’Malley, Christopher L. Fullerton, Alexis R. Mauger

**Affiliations:** 1 School of Sport and Exercise Sciences, University of Kent, Canterbury, Kent United Kingdom; 2 School of Kinesiology and Physical Activity Sciences, Université de Montréal, Montréal, Quebec Canada

**Keywords:** Perceived effort, Test–retest reliability, Psychophysiology, Cardiorespiratory, Affect

## Abstract

**Purpose:**

Using exercise protocols at a fixed rating of perceived effort (RPE) is a useful method for exploring the psychophysical influences on exercise performance. However, studies that have employed this protocol have arbitrarily selected RPE values without considering how these values correspond to exercise intensity thresholds and domains. Therefore, aligning RPE intensities with established physiological thresholds seems more appropriate, although the reliability of this method has not been assessed.

**Methods:**

Eight recreationally active cyclists completed two identical ramped incremental trials on a cycle ergometer to identify gas exchange threshold (GET). A linear regression model plotted RPE responses during this test alongside gas parameters to establish an RPE corresponding to GET (RPE_GET_) and 15% above GET (RPE_+15%GET_). Participants then completed three trials at each intensity, in which performance, physiological, and psychological measures were averaged into 5-min time zone (TZ) intervals and 30-min ‘overall’ averages. Data were assessed for reliability using intraclass correlation coefficients (ICC) and accompanying standard error measurements (SEM), 95% confidence intervals, and coefficient of variations (CoV).

**Results:**

All performance and gas parameters showed excellent levels of test–retest reliability (ICCs =  > .900) across both intensities. Performance, gas-related measures, and heart rate averaged over the entire 30-min exercise demonstrated good intra-individual reliability (CoV =  < 5%).

**Conclusion:**

Recreationally active cyclists can reliably replicate fixed perceived effort exercise across multiple visits when RPE is aligned to physiological thresholds. Some evidence suggests that exercise at RPE_+15%GET_ is more reliable than RPE_GET_.

**Supplementary Information:**

The online version contains supplementary material available at 10.1007/s00421-022-05094-z.

## Introduction

Perceived effort is a crucial determinant in the regulation of exercise intensity (Marcora 2008; Tucker 2009). In short, perceived effort is characterised as a psychophysiological phenomenon (Borg 1982) involving a complex interaction between physical stimuli (e.g., power/velocity) and perceptual responses (Gescheider 1997). Crucially, interpretations of perceived effort consider both subfactors. For instance, a lower perception of effort is denoted by an individual achieving a higher power/velocity for a given rating of perceived effort (RPE) value *or* a lower rating of effort for a given velocity/power.

Marcora (2009) highlights that perceived effort has two components, locomotor effort (Marcora et al. 2008) and respiratory effort (Dempsey et al. 2008). Locomotor effort encapsulates how hard, heavy, and strenuous the exercise task feels to drive the working muscles (Marcora 2010). Although it is still contested (see Pageaux 2016), effort perceptions surrounding locomotor effort are likely derived from the accumulation of central motor command by-products (e.g., corollary discharge) that are sent to working muscles (de Morree et al. 2012; Pageaux 2016). The accumulation of corollary discharge is believed to accumulate within cerebral centres such as the prefrontal cortex (de Morree et al. 2012) and anterior cingulate cortex (Pageaux et al. 2014; Meeusen and Roelands 2018) wherein perceptions of effort are generated.

Alternatively, respiratory effort is one of the perceptions associated with the multidimensional sensation of dyspnea (O’Donnell et al. 2009). Specifically, respiratory effort concerns the perception of how hard one is breathing (Laviolette and Laveneziana 2014). It is believed that respiratory effort originates within the brain’s anterior cingulate cortex where the efferent copies of motor command from respiratory muscles are centrally processed (Gigliotti 2010). Notably, the changes in the partial pressure of oxygen/carbon dioxide, and neuromuscular work of respiratory muscles may contribute towards the perceived difficulty to breathe (Amann et al. 2010; O’Donnell et al. 2020). Therefore, a combined model which acknowledges the combination of afferent feedback (e.g., chemical changes, breathing discomfort, and chest tightness) and perceptual/affective responses (e.g., inspiratory effort, unsatisfied inspiration) can help to explain the role of respiratory effort within the wider sensation of dyspnea (O’Donnell et al. 2020).

Borg’s 15-point RPE scale (Borg 1982) is widely accepted as the most convenient measure of assessing perceived effort. Initially conceived as a surrogate measure of exercise intensity/load (Borg 1982; Gescheider 1997), the use of the RPE scale has adapted to also allow contemporary researchers to obtain a singular gestalt value that simultaneously considers physical stimuli (i.e., velocity/power output), perceptual integration, and the individual inferences gleaned from the present context (Halperin and Emanuel 2020). In addition, the RPE scale (Borg 1982) and its derivatives (e.g., category-ratio 10 and 100, [Borg and Borg 2002]) have also been used to prescribe exercise intensity (Faulkner et al. 2007), quantify training load (Seiler and Kjerland 2006), and assess cardiorespiratory fitness (Faulkner et al. 2007; Mauger et al. 2013).

A novel method that has recently been employed is the use of fixed perceived effort exercise, during which individuals are required to exercise in accordance with their perceptions of effort (Cochrane et al. 2015a, b; Cochrane-Snyman et al. 2016, 2019; Astokorki and Mauger 2017a). Such a task is a unique opportunity for individuals to self-regulate their exercise whilst maintaining a fixed perceived intensity. Furthermore, recent studies (Cochrane et al. 2015a, b) have aligned RPE intensities with established physiological boundaries such as gas exchange threshold (GET) and respiratory compensation point (RCP). In doing so, researchers can begin to characterise the common psychophysiological response patterns that occur during fixed RPE exercise. Therefore, the procedure also allows researchers to examine the influence of additional psychophysiological phenomena (other than perceived effort) on exercise regulation within known intensity domains (Halperin and Emanuel 2020).

However, before implementing a specific protocol in practice, it is important for researchers to compare measures over repeated instances to determine whether they are reliable and that measures are precise. Across numerous laboratories, researchers, and studies, measured values should be accurately reproduced when the same procedure and measurements are repeated (Hopkins 2000). This concept is known as test–retest reliability and must apply to both inter (between individuals) and intra (within individual) levels with intraclass correlation coefficient (ICC) calculations determining whether a test is sufficiently reliable. Additionally, measures such as the standard error measurement (SEM) allow researchers to calculate the precision of these measurements and ascertain whether a substantial difference has occurred within subsequent studies that use the same methodology (Weir 2005).

Several studies have identified that fixed perceived effort activity is reliable. For instance, O’Grady et al. (2021) discerned that exercise at three separate RPE intensities was considered reliable at both the intra- and inter-individual level. Notably, the more intense the fixed effort exercise was, the more reproducible the findings were (i.e., RPE 17 demonstrated better reliability than RPE 9). Likewise, Cochrane-Snyman et al. (2016)—who utilised the more novel method of appropriating RPE intensities to known physiological boundaries—found that performance and electromyographic responses were consistent during 60-min fixed effort exercises. However, this study did not measure the cardiorespiratory markers despite the methodological aim to tailor RPE intensity to a known physiological boundary. Although a later study by the same group (Cochrane-Snyman et al. 2019) did investigate cardiorespiratory responses during fixed perceived effort exercise using this model, no results were presented to determine whether the cardiorespiratory responses were reliable.

Therefore, the purpose of the current study was to examine the test–retest reliability of three separate 30-min cycling trials whereby fixed perceived effort intensities were paired with exercising *at* (RPE_GET_) and *above* (RPE_+15%GET_) GET. This study tested two main hypotheses. First, both fixed perceived effort intensities would be consistently reproduced. Second, based on findings by previous studies (Eston and Williams 1988; Cochrane-Snyman et al. 2016; O’Grady et al. 2021), performance (e.g., power output [W]), physiological (e.g., heart rate [HR], relative oxygen uptake [$\dot{V}{\text{O}}_{{2}} .{\text{kg}}^{{ - {1}}}$], minute ventilation [$\dot{V}_{{\text{E}}}$], breathing frequency [BF]), and psychological (e.g., affect, self-efficacy) variables during a higher intensity fixed effort exercise would indicate higher reliability values compared to lower intensity fixed effort exercise.

## Methods

### Participants

Eight healthy (seven male; one female) recreationally active cyclists ([M ± SD] age: 24 ± 2.6 years; stature: 1.75 ± 0.1 m; mass: 72 ± 11.5 kg and maximum oxygen uptake [$\dot{V}{\text{O}}_{{2}} {\text{max}}$]: 54 ± 5.8 ml.kg^−1^.min^−1^) participated in the present study. All participants had at least 2 years of cycling experience (9 ± 3.4 years) and met nationally recognised guidelines for weekly physical activity (659 ± 386 min·wk^−1^). This met the level 3 classification from de Pauw et al. (2013). In addition, all participants were free from underlying cardiorespiratory or other pre-existing medical conditions and injuries that may have inhibited physical performance. None of the participants were currently taking any medication. Prior to providing written informed consent, participants were informed of the procedures, benefits, and risks of the study. The study was conducted in accordance with the principles of the Declaration of Helsinki and was approved by the School of Sport and Exercise Sciences Research Ethics Advisory Group (Prop 31_2019_20).

### Perceptual scales

In accordance with recent recommendations by Halperin and Emanuel (2020), the following steps were taken to ensure that the selection, use, and analysis of the RPE scale was adherent to maximising measurement validity. To reduce the ambiguity in the semantic representation of perceived effort, researchers provided a precise and consistent definition of perceived effort as “How hard, heavy and strenuous the exercise consciously feels to drive the working muscles and for your breathing” (Pageaux 2014). Throughout the study, the RPE scale was outlined with the same definition, instructions, and anchors on the 15-point Borg scale (1982) which participants rated their perceptions on. Alongside RPE, the 11-point Feeling Scale (Hardy and Rejeski 1989), measuring in-task affect, was incorporated to acknowledge similar phenomena such as discomfort and tiredness that may not be fully captured by the RPE scale alone. This use of the RPE scale was in accordance with the researchers’ collective ontological views.

The Feeling scale considered “How are you feeling at the present moment of the exercise?” on a scale from + 5 ‘I feel very good’ to − 5 ‘I feel very bad’. Finally, a single-item 11-point Likert scale questioned “How confident are you that you can tolerate the physical and mental effort associated with the cycling task”, with responses ranging from 0 ‘Not Confident at All’ to 10 ‘Extremely Confident’ with a mid-point of 5 ‘Moderately Confident’. This scale was adapted in line with Bandura’s (1997) framework. All scales were first explained during the recruitment process to participants.

### Experimental design

This study employed a within-participants randomised crossover design, wherein participants were required to visit the laboratory on eight separate occasions. All experimental sessions were conducted a minimum of 2 days and maximum of 7 days apart. Each participant’s visits were scheduled at the same time of day (± 2 h). *Visits 1 and 2* involved identical ramped incremental $\dot{V}{\text{O}}_{{2}} {\text{max}}$ tests on a cycle ergometer with an ensuing fixed effort familiarisation cycle. *Visits 3–8* consisted of 30-min fixed effort cycling bouts that matched to one of two intensities corresponding to RPE_GET_ and RPE_+15%GET_. Each condition was completed three times in a randomised fashion to prevent any order effects. Female participants completed each condition/intensity through one stage of menses (Luteal phase) to reduce any added confounding effects. After completion of all trials, participants were debriefed before being cleared to leave. All procedures took place in the same laboratory setting which had a constant temperate environment ([M ± SD] temperature, 19.3 ± 0.6 °C; humidity, 40.2 ± 4.3%; barometric pressure, 751.5 ± 3.2 mmHg). Participants were instructed to refrain from alcohol and intense exercise in the 48 h preceding testing and to abstain from caffeine consumption in the 4 h pre-testing. All testing took place at least 2 h after the last meal and participants were asked to replicate their eating habits before each session.

### Procedures

#### Visits 1 and 2: ramped incremental *V̇*O_2_max tests and familiarisations

Upon arrival to the laboratory, anthropometric data were obtained along with a 20 μl resting [La^−^]_b_ sample from the right-hand index finger which was lysed and assessed using an automated analyser (Biosen: C-Line, EKF Diagnostics, GmbH, Barleben, Germany). After this, participants were briefed on the protocols of the ramped incremental test, the scales used during the test, and subsequent familiarisation whilst being fitted with an HR monitor (Cyclus 2: ANT + , Leipzig, Germany) for measurements on a beat-by-beat basis. Participants were then asked to perform a short self-selected five-minute warm-up on the cycle ergometer (Cyclus 2, Leipzig, Germany) which allowed participants to mount their own bike frame for familiarity. Each participant used the same bike frame throughout all visits.

During the completion of the warm-up, the researcher re-explained the use and protocols concerning the RPE scale which would be administered throughout the test. After a completing the warm-up, participants were fitted with a mask that covered the nose and mouth and connected to a flowmeter that was attached to a metabolic cart system (Cortex Metalyser: Model 3B, Leipzig, Germany) which measured gas exchange parameters and pulmonary ventilation (inspired and expired flow rates) on a breath-by-breath basis. The gas analyser was pre-calibrated using a fixed 3-L syringe (Hans Rudolph, Kansas, USA) and known gas concentrations. After participants were fitted to the equipment, confirmed an understanding of the perceptual scales, and provided a resting value for the RPE scale, the ramped incremental test began. The affect and self-efficacy scales were used exclusively during the familiarisation and experimental trials.

For the ramped incremental tests, males were required to cycle at 80 W for 3 min to allow gas parameters to stabilise before commencing the test. Once elapsed, the incremental ramped test began at 100 W and increased incrementally by 25 W·min^−1^. In contrast, females were required to cycle at 40 W for three minutes to allow gas parameters to stabilise before the commencement of the *V̇*O_2_max test at 50 W with identical 25 W·min^−1^ ramped increments. These intensities were selected as pilot testing showed that these starting intensities and progressions resulted in all participants reaching volitional exhaustion within the recommended 8–10-min period (Yoon et al. 2007). All participants were informed to maintain a cadence above 80 revolutions·min^−1^ which should gradually increase as cycling intensity became harder until they could no longer sustain the exercise. Each minute (including at 50 [females] or 100 [males] W), RPE was recorded. Cardiorespiratory and power output were monitored continuously (each second) throughout the test. Participants were expected to perform to their maximum perceived ability. Whereupon the participant a) believed they had reached volitional exhaustion or b) cadence dropped below 60 revolutions·min^−1^ for more than 5 s despite strong verbal encouragement, the test was stopped. Additional RPE measures were taken at exhaustion alongside a final [La^−^]_b_ sample.

After the cessation of the ramped incremental test, participants received 15-min passive recovery and then conducted a 10-min familiarisation (5 min at RPE 13 and 15 each) to the fixed perceived effort cycling trials. During these familiarisation trials, participants maintained a cadence between 80 and 90 revolutions·min^−1^ which was then used as reference for the experimental visits. Intensities of RPE 13 and 15 were selected based on previous studies findings as to what RPE_GET_ and RPE_+15%GET_ correspond to (Cochrane et al. 2015b; Cochrane-Snyman et al. 2016).

#### Determination of RPE_GET_ and RPE_+15%GET_

Individual GETs were determined by utilising a *V̇*-slope method (Beaver et al. 1986) whereby GET corresponded to the point at which *V̇*O_2_ values above and below the breakpoint with *V̇*CO_2_ diverged from the intersection of the two linear regression lines. For validation, *V̇*-slope was used in conjunction with secondary criteria including: ventilatory equivalents; end-tidal volumes and respiratory exchange ratio. A secondary researcher was used to confirm that GET was assigned at the same place. Once GET was determined, *V̇*O_2_ values that were 15% above GET were also calculated. Using these values, the W that was exerted over the course of the ramped incremental test was plotted against the *V̇*O_2_ and a linear regression equation (y = m*x* + c) derived the W that corresponded to GET and 15% above GET. Finally, the ramped incremental power output data were plotted against the obtained RPE values in which an identical linear regression equation was used to identify RPE_GET_ and RPE_+15%GET_. These RPE values were rounded to the nearest whole number. An average of the two values from *Visits 1 and 2* was used as reference RPE points for *Visits 3–8*, experimental visits.

#### Fixed perceived effort cycling (experimental sessions)

After participants completed an identical warm-up and baseline measures to *Visits 1 and 2*, participants mounted the ergometer and were asked to cycle at RPE 10 (between “very light” and “light”) for 2 min. Once 2 min had elapsed, approximately 30–60 s was afforded for participants to ramp up to the required RPE intensity based on average times to reach the required RPE in pilot testing.

The researcher(s) stressed that the task was a fixed effort trial, meaning RPE must remain constant throughout. As a result, power output changes were expected; therefore, participants could change their power output by increasing/decreasing the virtual gears on the ergometer to ensure that the appropriate RPE was maintained throughout the entirety of the fixed effort cycles. It was advised that participants maintained a cadence between 80 and 90 revolutions min^−1^ throughout and that this cadence was replicated (± 2 revolutions·min−1) in all subsequent experimental visits.

**Fig. 1:**
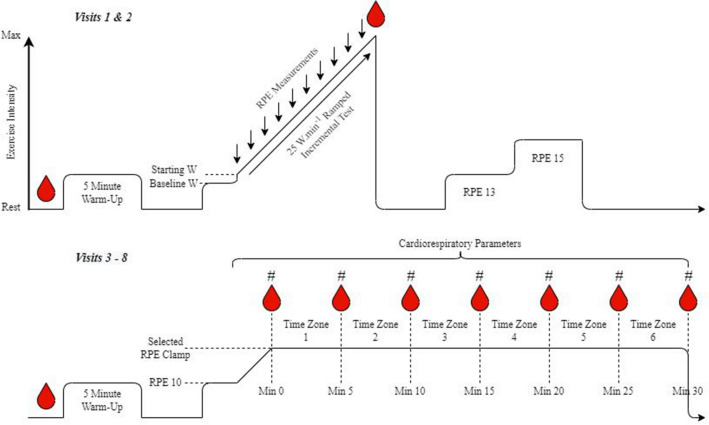
Representation of study protocols. Legend: # denotes affect and self-efficacy measurements

Throughout the fixed effort trials, all exercise-related data except cadence were screened from the participants to ensure that performance was appropriated according to a fixed perceived effort. Every 2 min, the researcher would reaffirm with the participant that exercise intensity was being tailored to the appropriate perceived effort rating. During fixed effort cycling, power output and cardiorespiratory markers were extracted continuously (each second) throughout the 30-min exercise. Every 5 min, including baseline (Minute 0), [La^−^]_b_, affective valence and self-efficacy were recorded. Fig. 1 depicts all testing procedures.

After the completion of all visits, participants were fully debriefed before being permitted to leave.

### Analysis

Continuous data (e.g., HR, gas parameters) from experimental session data were averaged into six discrete 5-min time zones (TZ) (e.g., TZ1 = average from Minute 00:00–Minute 04:59). Other data (e.g., [La^−^]_b_, perceptual measures) were grouped based on when they were extracted (e.g., minute 0, 5, etc.). Finally, all data were also averaged over the entirety of the exercise as ‘overall’ (average from Minute 0–Minute 30 or TZ1–TZ6).

All data were exported to SPSS (IBM: v.26, New York, USA) where data were assessed for normality and symmetry. Normality was assessed using the Shapiro–Wilk test and visual inspection of Q–Q plots before any subsequent analysis.

Power output, cardiorespiratory (e.g., HR, $\dot{V}{\text{O}}_{{2}} .{\text{kg}}^{{ - {1}}}$), and RPE responses from the ramped incremental tests were analysed according to 30-s averaged values. For *Visits 1 and 2*, a mean across both visits was calculated for values at peak, GET and 15% above GET. A single-measures, two-way random ICC (2,1) was calculated between both ramped incremental tests for peak, GET, and 15% above GET values with accompanying standard error measurements (SEM) to assess the test–retest reliability of *Visits 1 and 2*. ICC values were interpreted as > 0.9 excellent reliability, > 0.8 good reliability, > 0.6 questionable reliability and < 0.6 poor reliability A Pearson (*r*) correlation coefficient was also conducted to assess the relationship of performance (W), physiological (HR, $\dot{V}{\text{O}}_{{2}} .{\text{kg}}^{{ - {1}}}$), and psychometric (RPE) values between each ramped incremental test with values ≥ 0.9 indicating very strong, ≥ 0.8 strong, ≥ 0.6 moderate, ≥ 0.4 weak, and < 0.4 no association.

Test–retest (inter-individual) reliability for data within *Visits 3–8* (experimental sessions) was assessed across TZ averaged and ‘overall’ (30-min averaged) data for power output, HR, [La^−^]_b_ gas parameters ($\dot{V}{\text{O}}_{{2}} .{\text{kg}}^{{ - {1}}}$,$\dot{V}_{{\text{E}}}$, BF), and psychometric (affect and self-efficacy) data. When calculating reliability using a single-measures, two-way random ICC (2,1) and accompanying SEM, data from each visit within each condition were used. The SEM was used to calculate a minimal difference (see Eq. 1). Subsequent 95% confidence intervals (95% CI) for each of these variables were calculated by subtracting and adding the minimal difference to the group mean. A coefficient of variation (CoV) was also used to identify intra-individual variation for ‘overall’ 30-min averaged W, $\dot{V}{\text{O}}_{{2}} .{\text{kg}}^{{ - {1}}}$, HR, $\dot{V}_{{\text{E}}}$, BF, and [La^−^]_b_ with measurement errors of ≤ 5% indicative of reliability (Hopkins 2000; Tate and Klett 1959). As coefficients of variations were presented as percentages, the Tate and Klett (1959) method was used to calculate 95% CI for measures of intra-individual reliability (Weir 2005)

1 $${\text{Minimal Difference}}\, = \,{\text{SEM}} \, \times \,{1}.{96}\,\, \times \,\,\sqrt 2 .$$

A series of 2 $\times$ 6 repeated-measures ANOVAs were used to examine the condition and condition $\times$ time effects at every 5 min (TZ) for performance (W) and physiological (HR, $\dot{V}{\text{O}}_{{2}} .{\text{kg}}^{{ - {1}}}$, $\dot{V}_{{\text{E}}}$, and BF) variables between conditions. Similar 2 $\times$ 7 repeated-measures ANOVAs were used for [La^−^]_b_ and psychological (affect, self-efficacy) variables between conditions that were taken at every 5-min interval (min 0, 5, 10, etc.). Values for each TZ were taken as an average across all three visits. Averages of the three visits for 30-min ‘overall’ values were assessed for differences between conditions using a paired samples *t* test or non-parametric equivalent. Repeated-measures ANOVA tests used a Mauchley’s test wherein if sphericity was violated, a Greenhouse–Geisser adjustment was employed to the appropriate degrees of freedom to counter the increased risk of type one error. For all repeated-measures ANOVAs, significant main effects across condition and time were followed up with a one-way repeated-measures ANOVA and a subsequent Bonferroni post hoc test for specific TZ pairwise comparisons. Non-parametric equivalents (Friedman’s test, Wilcoxon signed-rank test) were used when data violated normality. An alpha level of *P* ≤ 0.05 was employed to assess statistical significance, whilst partial eta squared (${\eta }_{\mathrm{p}}^{2})$ provided an estimate of effect size of the ANOVAs (small = 0.01, medium = 0.10, large = 0.25). Any follow-up pairwise comparisons and *t* tests used a Cohen’s *d* calculation to determine effect size (≥  0.2 = small, ≥  0.5 = moderate, ≥  0.8 = large).

## Results

### Visits 1 and 2 (ramped incremental tests)

Correlation coefficient between visits: mean group data demonstrated a Peak W of 349 ± 36 W which showed a strong correlation between ramped incremental visits (ICC = 0.962, SEM = 6.97, *r* = 0.962). Mean peak $\dot{V}{\text{O}}_{{2}} .{\text{kg}}^{{ - {1}}}$ was 52 ± 7 mL.kg^−1^.min^−1^ and demonstrated a questionable correlation between ramped incremental trials (ICC = 0.792, SEM = 3.05, *r* = 0.925). Finally, mean peak HR was 194 ± 6 b.min^−1^ and demonstrated a strong correlation between ramped incremental trials (ICC = 0.916, SEM = 1.62, *r* = 0.945).

Mean W corresponding to GET was 201 ± 29 W and demonstrated a strong correlation between ramped incremental tests (ICC = 0.957, SEM = 6.01, *r* = 0.968). Mean $\dot{V}{\text{O}}_{{2}} .{\text{kg}}^{{ - {1}}}$ at GET was 33 ± 4 mL.kg^−1^.min^−1^ and demonstrated a strong correlation (ICC = 0.929, SEM = 1.12, *r* = 0.960). Finally, mean HR at GET was 158 ± 7 b.min^−1^ and demonstrated a questionable correlation between ramped incremental visits (ICC = 0.668, SEM = 4.14, *r* = 0.629).

Mean W corresponding to 15% above GET was 236 ± 34 W and demonstrated a strong correlation between ramped incremental trials (ICC = 0.955, SEM = 7.31, *r* = 0.963). Mean $\dot{V}{\text{O}}_{{2}} .{\text{kg}}^{{ - {1}}}$ at 15% above GET was 38 ± 5 mL.kg^−1^.min^−1^ and demonstrated a strong correlation between ramped incremental trials (ICC = 0.910, SEM = 1.49, *r* = 0.962). Finally, mean HR at 15% above GET was 168 ± 8 b.min^−1^ and demonstrated a questionable reliability between ramped incremental trials (ICC = 0.664, SEM = 4.36, *r* = 0.677).

Mean RPE at GET was 13.0 (13–somewhat hard). Mean RPE at 15% above GET was 14.7 (15–hard). Participant RPE values at GET ranged from 12 to 14, whilst RPE values at 15% above GET ranged from 14 to 16.

### Visits 3–8 (experimental sessions)

Test–retest reliability: Single measure test–retest reliability measures indicated that overall (30-min averaged) measures of W and $\dot{V}{\text{O}}_{{2}} .{\text{kg}}^{{ - {1}}}$ demonstrated an excellent degree of reliability within the RPE_GET_ condition (Table 1). Overall HR, [La^−^]_b_ (Table 1), $\dot{V}_{{\text{E}}}$ (ICC = 0.839, SEM = 5.08), and self-efficacy (ICC = 0.807, SEM = 0.45) measures showed a good degree of reliability, whilst overall BF (ICC = 0.728, SEM = 1.66) and affect (ICC = 0.749, SEM = 0.48) showed a questionable reliability within the RPE_GET_ condition Within the RPE_+15%GET_ condition, overall measures of W, $\dot{V}{\text{O}}_{{2}} .{\text{kg}}^{{ - {1}}}$, [La^−^]_b_ (Table 2), $\dot{V}_{{\text{E}}}$ (ICC = 0.963, SEM = 3.26), and BF (ICC = 0.969, SEM = 0.96) demonstrated an excellent degree of reliability, whilst HR showed a good degree of reliability (Table 2), and affect (ICC = 0.770, SEM = 0.65), and self-efficacy (ICC = 0.711, SEM = 0.65) demonstrated questionable reliability. Main group mean overall and TZ results can be seen in Tables 1 and 2. Additional tables concerning $\dot{V}_{{\text{E}}}$, BF, affect, and self-efficacy can be found in supplementary materials.

**Table 1:** Group mean RPE_GET_ inter- and intra-individual results for each time zone and overall

Variable	TZ	Mean	SD	ICC (2,1)	SEM	95% CI	*CoV*
W	1	184	8.1	0.903	2.5	177–192	4.4
	2	182	8.0	0.919	2.3	176–188	
	3	179	7.3	0.924	2.0	174–185	
	4	176	8.4	0.906	2.6	169–184	
	5	176	9.7	0.884	3.3	166–184	
	6	175	9.8	0.887	3.3	166–184	
	Overall	179	8.0	0.915	2.3	172–185	
HR	1	144	8.8	0.566	5.8	128–160	3.1
	2	153	12.4	0.882	4.2	142–165	
	3	155	13.2	0.884	4.5	143–168	
	4	156	12.6	0.806	5.5	141–171	
	5	157	12.7	0.778	6.0	141–174	
	6	158	13.0	0.805	5.8	142–174	
	Overall	154	11.9	0.825	5.0	140–168	
$\dot{V}{\text{O}}_{{2}} .{\text{kg}}^{{ - {1}}}$	1	33	5.5	0.915	1.6	29–38	4.2
	2	35	6.7	0.950	1.5	31–39	
	3	35	6.9	0.943	1.7	30–40	
	4	35	7.1	0.921	2.0	29–40	
	5	35	7.3	0.928	2.0	29–40	
	6	35	7.6	0.910	2.3	29–41	
	Overall	35	6.8	0.932	1.8	30–40	
[La^−^]_b_	Min 0	2.46	0.6	0.735	0.3	1.55–3.37	12.7
	Min 5	3.63	1.3	0.837	0.5	2.21–5.04	
	Min 10	4.04	1.9	0.820	0.8	1.85–6.23	
	Min 15	4.24	2.2	0.881	0.8	2.10–6.37	
	Min 20	4.10	2.1	0.823	0.9	1.61–6.60	
	Min 25	4.05	2.3	0.835	0.9	1.51–6.59	
	Min 30	4.20	2.6	0.831	1.1	1.26–7.14	
	Overall	3.34	1.6	0.849	0.6	1.67–5.01

**Table 2:** Group mean RPE_+15%GET_ inter- and intra-individual results for each time zone and overall

Variable	TZ	Mean	SD	ICC (2,1)	SEM	95% CI	*CoV*
W	1	219	10.9	0.896	3.52	209–229	2.2
	2	208	5.0	0.941	1.22	205–212	
	3	201	7.0	0.928	1.89	195–206	
	4	199	4.7	0.945	1.11	196–202	
	5	195	4.8	0.960	0.95	193–198	
	6	193	5.5	0.943	1.32	190–197	
	Overall	203	4.3	0.962	0.84	201–206	
HR	1	159	9.0	0.807	3.97	148–170	1.6
	2	167	10.5	0.849	4.10	156–179	
	3	168	11.1	0.853	4.24	156–180	
	4	169	10.4	0.874	3.70	159–179	
	5	170	11.0	0.853	4.22	158–182	
	6	171	11.9	0.868	4.31	159–183	
	Overall	167	10.5	0.876	3.69	157–178	
$\dot{V}{\text{O}}_{{2}} .{\text{kg}}^{{ - {1}}}$	1	39	5.5	0.902	1.73	34–44	2.7
	2	40	6.1	0.947	1.40	37–44	
	3	39	6.1	0.931	1.59	35–44	
	4	39	6.0	0.939	1.47	35–43	
	5	39	6.4	0.937	1.62	35–43	
	6	39	6.5	0.936	1.64	34–43	
	Overall	39	6.0	0.951	1.34	36–43	
[La^−^]_b_	Min 0	3.36	0.9	0.813	0.4	2.28–4.44	9.2
	Min 5	6.25	2.2	0.819	0.9	3.68–8.82	
	Min 10	6.95	2.9	0.871	1.0	4.07–9.84	
	Min 15	6.76	3.2	0.948	0.7	4.74–8.79	
	Min 20	6.86	3.5	0.941	0.8	4.51–9.20	
	Min 25	6.85	3.8	0.953	0.8	4.58–9.11	
	Min 30	6.70	3.8	0.917	1.1	3.69–9.72	
	Overall	5.47	2.4	0.939	0.6	3.80–7.13	

When assessing 5-min TZ data, W reliability within the RPE_GET_ condition was excellent from TZ1–4, whilst TZ5–6 were considered good. Within the RPE_+15%GET_ condition, all time zones except TZ1 indexed an excellent degree of reliability.

During the RPE_GET_ and RPE_+15%GET_ condition, all $\dot{V}{\text{O}}_{{2}} .{\text{kg}}^{{ - {1}}}$ values demonstrated an excellent degree of reliability across all time zones. During the RPE_GET_ condition, HR values showed a good degree of reliability within TZ2, 3, 4, and 6, whilst TZ5 showed questionable reliability and TZ1 showed poor reliability. Alternately, within the RPE_+15%GET_ condition, all HR TZ data showed a good degree of reliability.

During the RPE_GET_ condition, $\dot{V}_{{\text{E}}}$ showed good reliability across all time zones (ICC = 0.801–0.871, SEM = 3.54–6.92) except TZ5 which showed questionable reliability (ICC = 0.778, SEM = 6.78). During the RPE_+15%GET_ condition, excellent reliability across all time zones (ICC = 0.933–0.951, SEM = 4.03–5.27) was observed except at TZ1 which showed good reliability (ICC = 0.827, SEM = 4.76). During the RPE_GET_ condition, BF showed questionable validity across all time zones (ICC = 0.640–0.776, SEM = 1.37–2.15), whereas the RPE_+15%GET_ condition showed excellent reliability across all time zones (ICC = 0.903–0.961, SEM = 1.21–1.85) except TZ1 which showed good reliability (ICC = 0.889, SEM = 1.31).

During the RPE_GET_ condition, [La^−^]_b_ demonstrated good reliability at every timepoint except minute 0 (questionable) (Table 1), whereas the RPE_+15%GET_ condition demonstrated excellent reliability of measures taken at minute 15–30 and good reliability at measures taken from minute 0–10 (Table 2).

During the RPE_GET_ condition, affect demonstrated good reliability at minute 0–5 (ICC = 0.831 and 0.826, SEM = 0.53 and 0.45), questionable reliability at minute 10, 15, and 25 (ICC = 0.686–0.786, SEM = 0.41–0.68), and poor reliability at minute 20 and 30 (ICC = 0.597 and 0.488, SEM = 0.69 and 0.81). During the RPE_+15%GET_ condition, affect demonstrated questionable reliability from minute 0–15 and minute 30 (ICCs = 0.621–0.720, SEM = 0.80–0.95), and poor reliability at minute 20–25 (ICCs = 0.552–0.592, SEM = 0.79–0.95).

Self-efficacy data during the RPE_GET_ condition demonstrated good reliability at minute 0, 5, and 30 (ICCs = 0.812–0.883, SEM = 0.43–0.63), questionable reliability at minute 10–20, (ICCs = 0.636–0.765, SEM = 0.59–0.63), and poor reliability at minute 25 (ICC = 0.505, SEM = 0.57). Self-efficacy data during the RPE_+15%GET_ condition demonstrated a good reliability at minute 0 and 5 (ICCs = 0.850 and 0.815, SEM = 0.75 and 0.77), questionable reliability at minute 10 (ICC = 0.607, SEM = 0.99), and poor reliability at minute 15–30 (ICCs = 0.427–0.524, SEM = 0.84–0.99).

Intra-individual reliability: Measures of intra-individual reliability demonstrated that overall W varied by a mean ± SD of 4.4 ± 1.5% (95% CI 2.9–8.9%) within the RPE_GET_ condition, whereas the RPE_+15%GET_ condition varied by 2.2 ± 1.1% (95% CI 1.5–4.5%) on average.

Overall $\dot{V}{\text{O}}_{{2}} .{\text{kg}}^{{ - {1}}}$ was 4.2 ± 1.5% (95% CI 2.8–8.5%) during the RPE_GET_ condition and 2.7 ± 1.3% (95% CI 1.8–5.5%) during the RPE_+15%GET_ condition. Variability in Overall HR was 3.1 ± 1.1% (95% CI 2.0–6.2%) in the RPE_GET_ condition and 1.6 ± 1.2% (95%CI 1.1–3.3%) in the RPE_+15%GET_ condition.

Mean ± SD overall $\dot{V}_{{\text{E}}}$ variability was 6.2 ± 1.2% (95% CI 3.2–9.3) during the RPE_GET_ condition and 2.8 ± 1.1% (95% CI 1.0–4.6) during the RPE_+15%GET_ condition. Overall BF variability was 4.0 ± 2.0% (95% CI 3.1–5.0) during the RPE_GET_ condition and 2.6 ± 1.1% (95% CI 1.9–3.3) during the RPE_+15%GET_ condition. Mean ± SD overall [La^−^]_b_ variability was 12.7 ± 9.6% (95% CI 12.4–13.0) during the RPE_GET_ condition and 9.2 ± 7.3% (95% CI 8.9–9.4) during the RPE_+15%GET_ condition.

Differences between RPE_GET_ and RPE_+15%GET_ conditions and time zones: A series of 2 $\times$ 6 repeated-measures ANOVAs determined significantly large condition effects for W, HR, $\dot{V}{\text{O}}_{{2}} .{\text{kg}}^{{ - {1}}}$, $\dot{V}_{{\text{E}}}$, and BF measures (*F* = 43.377–69.336, *P* = 0.001–0.002, ${\eta }_{\rho }^{2}$ = 0.861–0.908). Significantly large condition $\times$ time effects were observed for W, $\dot{V}{\text{O}}_{{2}} .{\text{kg}}^{{ - {1}}}$, and BF (*F* = 4.950–6.609, *P* = 0.002–0.007, ${\eta }_{\rho }^{2}$ = 0.366–0.486).

A series of 2 $\times$ 7 repeated-measures ANOVAs determined significantly large condition effects for [La^−^]_b_, affect, and self-efficacy measures (*F* = 19.505–59.163, *P* = 0.001–0.003, ${\eta }_{\rho }^{2}$ = 0.736–0.894). Significantly large condition $\times$ time effects were observed for [La^−^]_b_ and affect (*F* = 6.811–10.241, *P* = 0.001–0.017, ${\eta }_{\rho }^{2}$ = 0.493–0.594).

Additional one-way repeated-measures ANOVAs determined significant changes over time in W, HR, and BF during the RPE_GET_ condition (*F* = 5.530–20.494, *P* = 0.001–0.017). Significant changes over time were observed for W, HR, BF, [La-]b, and affect during the RPE_+15%GET_ condition (*F* = 6.485–28.295, *P* = 0.001–0.031).

**Fig. 2:**
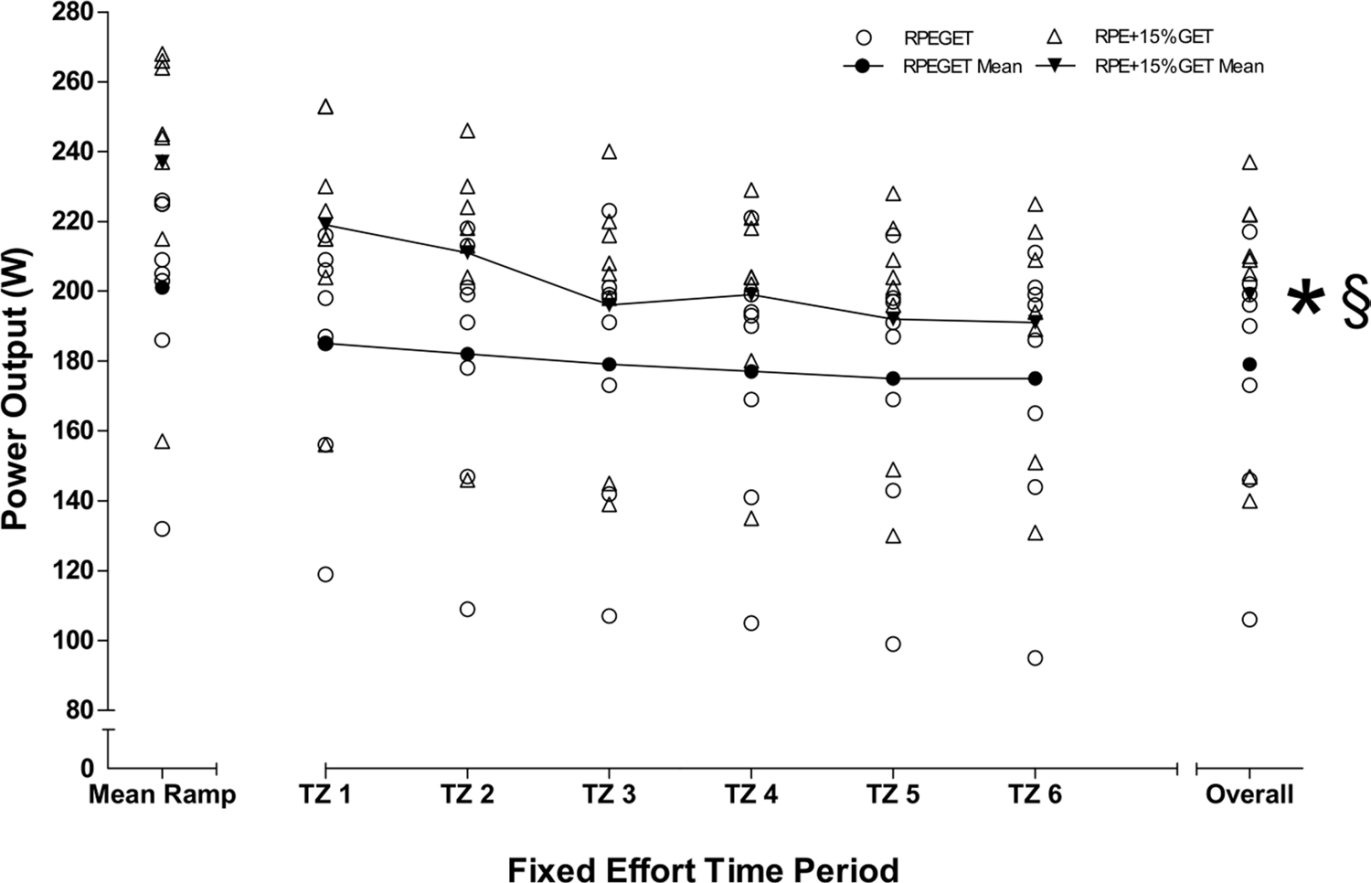
Mean ± SD across all three condition experimental visits in time-lapsed changes in W at each 5-min TZ and overall, during the 30-min fixed effort cycling exercise. Legend: * denotes a significant difference in overall values between conditions (*P* < .05), § denotes a moderate effect size

**Fig. 3:**
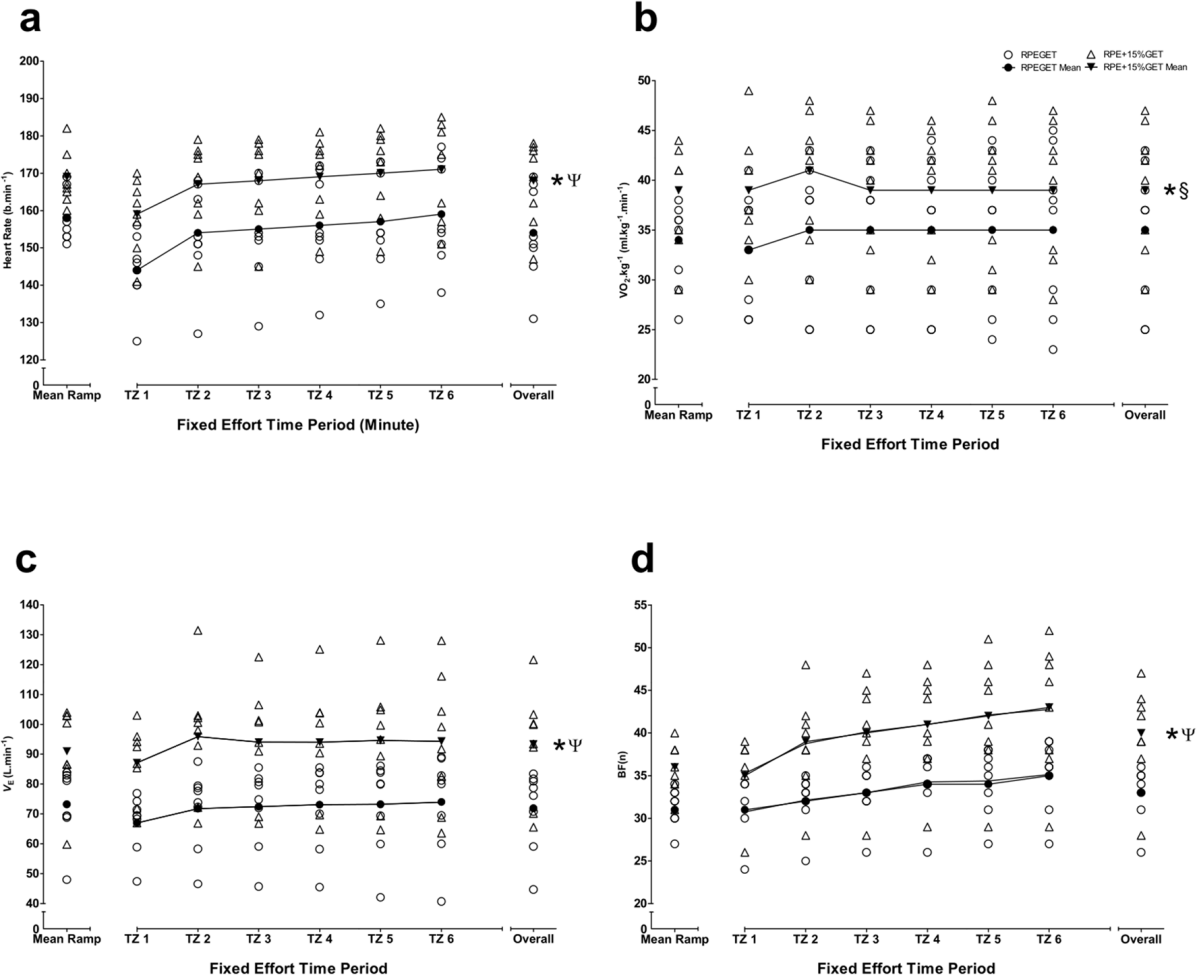
Mean ± SD across all three condition experimental visits in time-lapsed changes in cardiorespiratory parameters (**a** = HR, **b** = *V̇*O_2_.kg^−1^, **c** = *V̇*_E_, **d** = BF) at each five-minute TZ and overall, during the 30-min fixed effort cycling exercise. Legend: * denotes a significant difference in overall values between conditions (*P* < .05), § denotes a moderate effect size, and Ψ denotes a large effect size

**Fig. 4:**
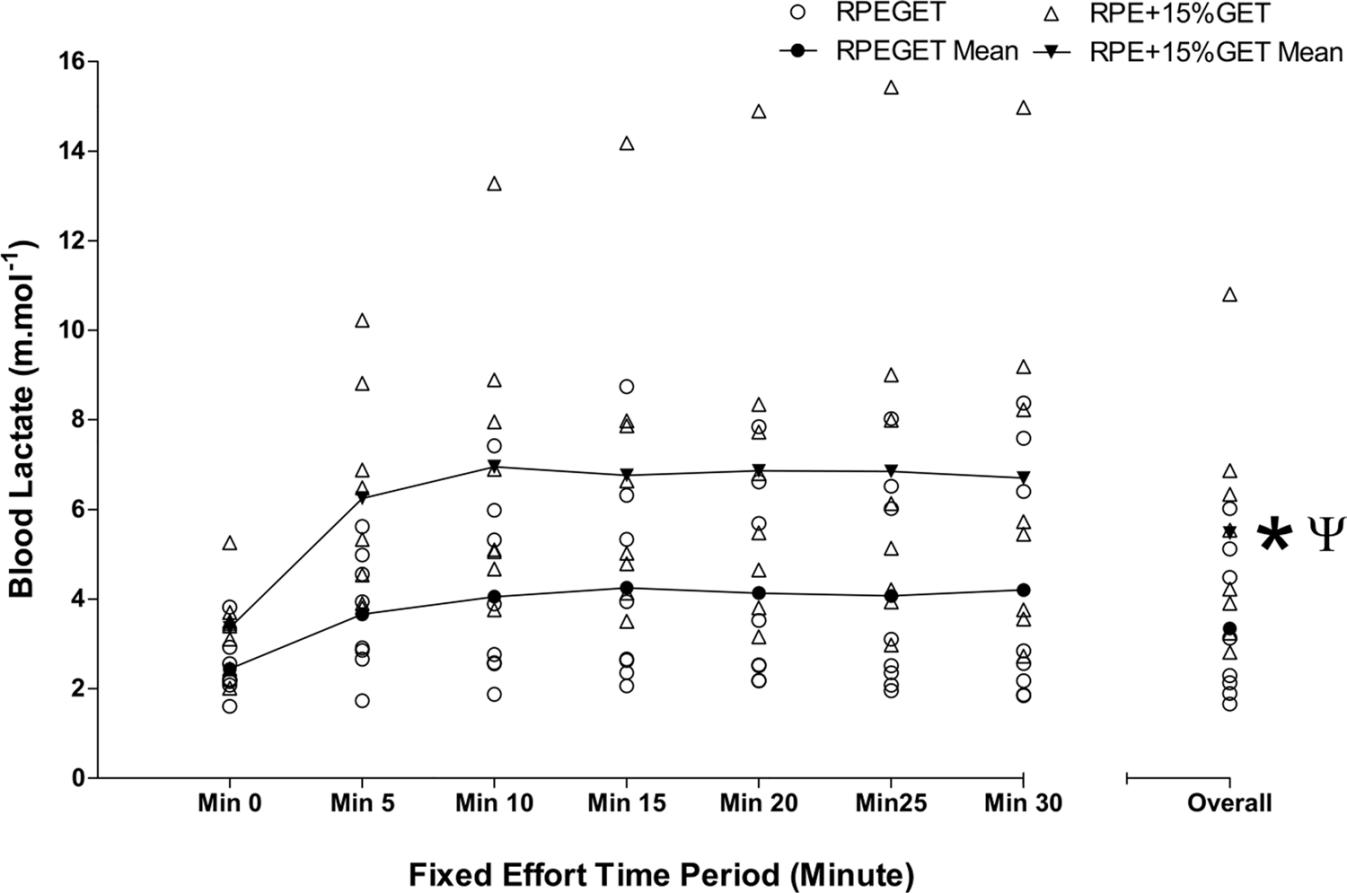
Mean ± SD across all three condition experimental visits in time-lapsed changes in [La^−^]_b_ at each 5-min timepoint and overall, during the 30-min fixed effort cycling exercise. Legend: * denotes a significant difference in overall values between conditions (*P* < .05), § denotes a moderate effect size, and Ψ denotes a large effect size

**Fig. 5:**
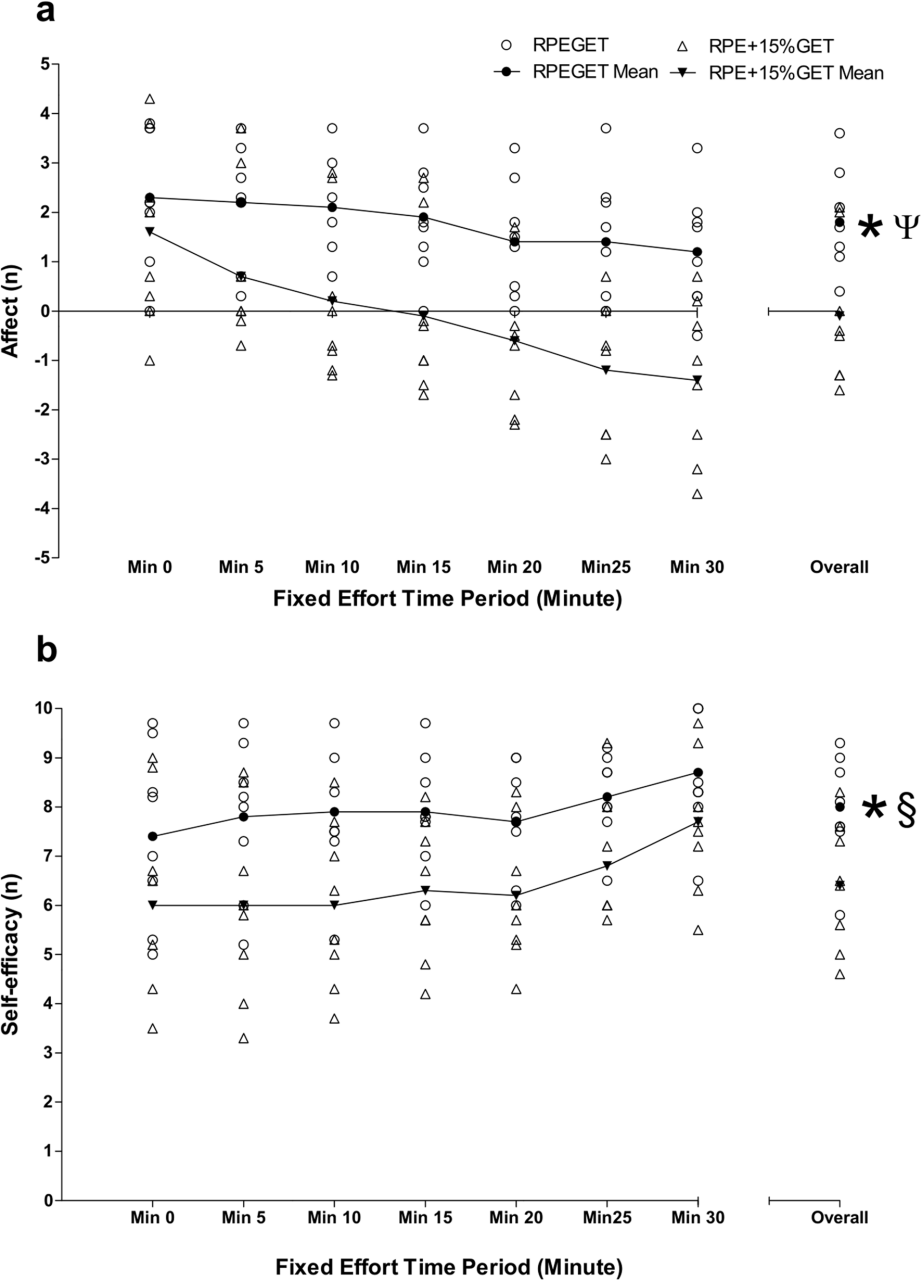
Mean ± SD across all three condition experimental visits in time-lapsed changes in psychological parameters: **a** = affective valence, **b** = self-efficacy at each 5-min timepoint and overall, during the 30-min fixed effort cycling exercise. Legend: * denotes a significant difference in overall values between conditions (*P* < .05), § denotes a moderate effect size, and Ψ denotes a large effect size

During the RPE_GET_ condition, follow-up Bonferroni corrected post hoc analyses revealed significant differences in HR at TZ1 and 4–6 (*P* = 0.019–0.023) and TZ2 and 3 (*P* = 0.018), and BF at TZ1–2 and 4 (*P* = 0.029–0.042). During the RPE_+15%GET_ condition, Bonferroni post hoc analyses determined significant differences in: W at TZ1 and 3–6 (*P* = 0.006–0.024) and TZ2 and 3–6 (*P* = 0.003–0.025); HR at TZ1 and 2–6 (*P* = 0.010–0.025); $\dot{V}{\text{O}}_{{2}} .{\text{kg}}^{{ - {1}}}$ at TZ2 and 3–4 (*P* = 0.001–0.018); BF at TZ2 and 5 (*P* = 0.024); and affect at minute 0–20 and minute 30 (*P* = 0.036–0.050). Overall W, HR, $\dot{V}{\text{O}}_{{2}} .{\text{kg}}^{{ - {1}}}$, BF, [La^−^]_b_, and self-efficacy were significantly different between conditions (*t* = 4.362–8.497,* P* = 0.001–0.003). Overall $\dot{V}_{{\text{E}}}$ and affect were significantly different between conditions (*Z* = 2.524–2.527,* P* = 0.012). Large effect sizes were observed for HR, $\dot{V}_{{\text{E}}}$, BF, [La^−^]_b_, affect, and self-efficacy (d = 1.00–1.58). Moderate effect sizes were observed for W and $\dot{V}{\text{O}}_{{2}} .{\text{kg}}^{{ - {1}}}$ (*d* = 0.58–0.75). Figures 2, 3, 4, 5 depict the changes of three visit averages in performance, physiological, and psychological during the fixed perceived effort trials.

## Discussion

The present study aimed to assess the test–retest reliability of 30-min fixed perceived effort cycling trials which used a linear regression model to fix RPE intensity according to physiological thresholds. Foremostly, results showed that 30-min fixed effort cycling demonstrated good test–retest and intra-individual reliability amongst a cohort of recreationally active cyclists. This was supported by ICC values which evidenced that overall performance measures (e.g., W) demonstrated an excellent degree of reliability (> 0.900) between visits in both conditions. In addition, overall physiological variables, such as $\dot{V}{\text{O}}_{{2}} .{\text{kg}}^{{ - {1}}}$, $\dot{V}_{{\text{E}}}$, BF, and [La^−^]_b_, also demonstrated an excellent degree of reliability (> 0.900) in the RPE_+15%GET_ condition. Test–retest reliability for HR demonstrated good reliability (> 0.800) across both conditions.

Other research has also exhibited that perception of effort remains consistent over different exercise tasks such time-to-exhaustion trials (Okuno et al. 2015) and time-trials (Borg et al. 2018). Furthermore, irrespective of exercise modality, previous studies (Cochrane et al. 2015a, b; Eston and Williams 1988) have identified that fixed perceived effort exercise can be reliably replicated across visits. Such findings are consistent with those observed in this study as measures of performance (W) and physiological response ($\dot{V}{\text{O}}_{{2}} .{\text{kg}}^{{ - {1}}}$,$\dot{V}_{{\text{E}}}$, BF, and [La^−^]_b_) showed excellent measures of test–retest reliability (ICC =  > 0.900 with small < 6% SEM from the group mean) (Weir 2005). Therefore, it appears that recreationally active athletes can consistently reproduce physical efforts that are regulated by perceptions alone. This may be beneficial for practitioners and coaches alike in the future who lack the resources to measure intricate psychophysical markers that relate to specific workloads and physiological thresholds. Instead, RPE can be used as a surrogate measure during physical activity.

In addition, the present study also assessed intra-individual reliability measures, in which participants demonstrated low CoV values (≤ 5%) and narrow 95% CI for overall performance (W) and physiological ($\dot{V}{\text{O}}_{{2}} .{\text{kg}}^{{ - {1}}}$, HR, $\dot{V}_{{\text{E}}}$, and BF) variables. However, it was notable that [La^−^]_b_ varied significantly (12.7% in RPE_GET_ and 9.2% in RPE_+15%GET_). This finding may discredit the use of lactate as a reliable indicator of exercise intensity if variations between individuals exist so prominently. For instance, the use of maximal lactate steady state has come under increased scrutiny in recent years as opposed to other mathematical models to determine maximal aerobic capacity (Jones et al. 2019). As such, these arguments may be further validated by the findings of the current study.

As noted, only one study to date (Cochrane-Snyman et al. 2016) has explored the reliability of performance and physiological parameters during a fixed effort exercise in which RPE has been tailored to known physiological thresholds/domains. However, this study only utilised correlation coefficients and ICCs to assess the reliability of repeated fixed effort performance, despite research advocating that 95% confidence intervals are a more robust alternative (Hopkins 2000). At the intra-individual level, participants of the present study were able to replicate their efforts consistently between visits in both the RPE_GET_ and RPE_+15%GET_ condition. Moreover, the 95% CI for most participants remained below 5% to further substantiate this conviction. Paton and Hopkins (2001) identified that self-paced cycling trials usually produce variances of 2–3%. The findings of the current study—particularly data in the RPE_+15%GET_ condition—remain close to this range of variances as PO, $\dot{V}{\text{O}}_{{2}} .{\text{kg}}^{{ - {1}}}$, and HR demonstrated CoVs between 3.1 and 4.4% in the RPE_GET_ condition, and 1.6–2.7% in the RPE_+15%GET_ condition.

Many have ascribed this consistency in performance to the athlete’s familiarity (i.e., experience level, practice) to the exercise tasks. With this is mind, several factors can help rationalise why this study showed the degree of reliability it did, and subsequently inform future research studies to obtain similarly reliable and comparable data. First, the participants that were recruited within this study were all healthy, active, and experienced cyclists. In doing so, this likely led to a more homogenous sample which has consequences for the reliability measures that are calculated (Hopkins 2000). All participants demonstrated very good-to-excellent physiological measures (e.g., $\dot{V}{\text{O}}_{{2}} {\text{max}}$, %$\dot{V}{\text{O}}_{{2}} {\text{max}}$ at GET) during the ramped incremental trials (de Pauw et al. 2013). Therefore, having a collection of participants with a narrower distribution of physiological capabilities compared to other studies (Cochrane et al. 2015a; Bergstrom et al. 2015) could explain the low CoV values and confidence intervals observed in this study.

In addition, as all participants were trained, albeit recreationally, it may be assumed that participants in this study were more attuned to the underlying physiological signals (Elferink-Gemser and Hettinga 2017) during the fixed effort trials compared to previous studies that have used less-trained cohorts (e.g., Cochrane et al. 2015a). Notably, this study involved fixed effort exercise which was aligned to known physiological thresholds, such as GET. Thus, a cohort of currently active individuals who are aware of the typical physiological sensations and perceptions associated with such thresholds could mean that it became substantially easier to taper their efforts according to the RPE value itself as well as the physiological sensations associated with that RPE (Lamb et al. 1999).

Moreover, another critical factor to the reliability of this study could have been the employment of multiple familiarisation trials. Conducting exercise at a fixed RPE is a relatively artificial exercise task; therefore, the opportunity for participants to familiarise themselves twice before the experimental trials could be a key factor. Extant literature has evidenced that the inclusion of familiarisation trials significantly improves the validity and reproducibility of performance indices during self-regulated RPE-based exercise (Lim et al. 2016). Furthermore, Mauger et al. (2014) determined that a cohort active males could replicate fixed effort exercises even without reference to the scale, relying solely on internal psychophysical sensations due to previous experience.

Another notable finding of this study was that RPE_+15%GET_ results demonstrated much lower variability at both the inter- and intra-individual levels compared to the RPE_GET_ condition. A previous study by O’Grady et al. (2021) determined that fixed effort exercise at higher RPE values rendered lower between and within individual variances in power output and cardiorespiratory parameters compared to fixed effort exercise at lower RPE values. In addition, other studies appear to share similar conclusions based on their results (Eston and Williams 1988; Cochrane-Snyman et al. 2016). However, it was not explained why harder intensity fixed effort exercise appears to be better replicated than lower intensity fixed effort exercise.

One possible suggestion is that during harder intensity exercise, participants may employ different methods of decision-making according to the different physiological sensations associated with harder intensity compared to lower intensity exercise (Renfree et al. 2014). To illustrate, when exercising at RPE_+15%GET_, participants usually begin exercising within the heavy intensity domain (Gaesser and Poole 1996). Whilst in this domain, athletes experience growing levels of metabolites (e.g., H + ions), nociceptive stimulation (Mauger 2014), and afferent feedback (Amann et al. 2009). As a result, Renfree et al. (2014) suggest that this may engender athletes to adopt more heuristic decision-making processes. This is because the overbearing discomfort and negatively oriented sensations/perceptions—as seen in this study (Fig. 5)—that arise due to harder intensity exercise may cause athletes to make decisions based on more select pieces of information to save effort (Gigerenzer and Gaissmaier 2011). Therefore, responses become more ‘primal’ and ‘instinctive’, meaning that they may be more easily replicated as they are based on stable trait-like factors.

On the other hand, exercise at RPE_GET_ is expected to occur entirely within the moderate intensity domain whereby metabolite production equals metabolite clearance (Gaesser and Poole 1996). Therefore, the athlete experiences fewer negative sensations and perceptions, such as discomfort and pain. Consequently, Renfree et al. (2014) suggest that this would endear the athlete to employ more rational-based decision-making. As a result, more situational factors are considered when regulating exercise intensity, which could translate into more variances in behaviour overall. However, as this study did not monitor the underlying decision-making processes during the fixed effort exercise, firmer conclusions cannot be drawn. Nonetheless, recent studies have employed the use of a novel “Think-Aloud” protocol which allows researchers to understand the underlying thought and decision-making processes that are articulated during an endurance event (Whitehead et al. 2018). In line with this, future research may wish to consider the use of Think-Aloud approaches to begin to discern how effort is consciously regulated and the concomitant changes to psychophysiological processes as a result.

Finally, it is interesting to note the differences in the trajectory of responses between conditions during this study. Although the study aims primarily focussed on the reliability measures associated with novel fixed perceived effort cycling trials, some discussion can also be generated around the potential mechanisms that underpin the changes in performance, physiological, and psychological indices that were measured in this study. For instance, all performance (W), physiological (HR, $\dot{V}{\text{O}}_{{2}} {\text{max}}$, $\dot{V}_{{\text{E}}}$, BF, [La^−^]_b_), and psychological (affect and self-efficacy) measures were significantly different between conditions at all TZ/time points and overall. In particular, responses for affect were negative throughout the entire fixed effort exercise in the RPE_+15%GET_ condition compared to a gradual decrease from positive to neutral in the RPE_GET_ condition (Fig. 5).

Numerous studies have highlighted that affective valence may be a useful indicator of future exercise uptake and adherence (Brand and Ekkekakis 2021). To illustrate, studies have exhibited that when individuals completed exercise in line with a positive affect (Parfitt et al. 2012a), individuals were more likely to continue engaging in exercise compared to a fixed power output/velocity exercise. Interestingly, this was despite there being no actual differences in the actual physical intensity of the exercise between conditions (Parfitt et al. 2012a, b). Results from these studies demonstrate that a fixed effort exercise at lower RPE values (e.g., RPE_GET_) is reliable and elicits more positive/neutral affective responses may provide a useful method for future studies focussing on exercise prescription and adherence.

## Conclusion

Overall, this study has demonstrated that recreationally active cyclists can execute reliable fixed effort exercise cycling trials which are aligned to physiological thresholds/domains. It appears that the harder the RPE intensity, the more reliably exercises can be conducted at both within and between individual levels. However, the underpinning factors for this remain unknown and yet to be fully explored. Some possible avenues for exploration may be the underlying decision-making processes that influence exercise behaviours during fixed effort cycling. Finally, this study also noted a significant difference in all performance, physiological, and psychological variables between conditions. Notably, affect was continually negative throughout the more intense RPE_+15%GET_ compared to the less-intense RPE_GET_ condition. This may be of benefit to studies within the exercise rehabilitation domain as comparative findings suggest exercising at lower fixed perceived intensities that maintain positive affect may be better for exercise uptake and adherence. However, a continued exploration of this topic is required.

## Supplementary Information

Below is the link to the electronic supplementary material.

Supplementary file1 (DOCX 22 KB)

## Data Availability

Raw data are available upon request from the corresponding author.

## Abbreviations

[La^−^]_b_: Blood lactate
ANOVA: Analysis of variance
BF: Breathing frequency
CoV: Coefficient of variation
CI: Confidence interval
GET: Gas exchange threshold
HR: Heart rate
ICC: Intraclass correlation coefficient
RCP: Respiratory compensation point
RPE: Ratings of perceived effort
RPE_+15%GET_: Ratings of perceived effort at 15% above gas exchange threshold
RPE_GET_: Ratings of perceived effort at gas exchange threshold
SEM: Standard error measurement
TZ: Time zone
$\dot{V}{\text{CO}}_{{2}}$: Carbon dioxide production (absolute)
$$\dot{V}_{{\text{E}}}$$: Minute ventilation
$\dot{V}{\text{O}}_{{2}} .{\text{kg}}^{{ - {1}}}$: Oxygen uptake (relative)
$\dot{V}{\text{O}}_{{2}} {\text{max}}$: Maximum oxygen uptake
W: Power output
