# Supplementary material for: Test–retest reliability of a 30-min fixed perceived effort cycling exercise
Source: Eur J Appl Physiol. 2022 Nov 27;123(4):721–35. doi: 10.1007/s00421-022-05094-z (PMC10030391; doi:10.1007/s00421-022-05094-z)
Supplement: Supplementary file 1 — Supplementary file1 (DOCX 22 KB) [file 421_2022_5094_MOESM1_ESM.docx]

| Supplementary Table 1. Group mean RPE_GET_ inter- and intra-individual results for each time zone and overall. | | | | | | | |
| --- | --- | --- | --- | --- | --- | --- | --- |
| Variable | TZ | Mean | SD | ICC (2,1) | SEM | 95% CI | *CoV* |
| *V̇*_E_ | 1 | 67.0 | 8.9 | 0.841 | 3.54 | 57.2 – 76.2 | *6.2* |
|  | 2 | 71.8 | 12.3 | 0.868 | 4.46 | 59.4 – 84.2 |  |
|  | 3 | 72.4 | 12.6 | 0.871 | 4.53 | 59.8 – 85.0 |  |
|  | 4 | 73.1 | 13.5 | 0.812 | 5.83 | 56.9 – 89.3 |  |
|  | 5 | 73.2 | 14.4 | 0.778 | 6.78 | 54.4 – 92.1 |  |
|  | 6 | 74.0 | 15.5 | 0.801 | 6.92 | 54.8 – 93.2 |  |
|  | Overall | 71.9 | 12.6 | 0.839 | 5.08 | 57.9 – 86.0 |  |
| BF | 1 | 31 | 2.9 | 0.776 | 1.37 | 27 – 35 | *4.0* |
|  | 2 | 32 | 3.1 | 0.698 | 1.71 | 27 – 37 |  |
|  | 3 | 33 | 3.1 | 0.726 | 1.61 | 28 – 37 |  |
|  | 4 | 34 | 3.4 | 0.715 | 1.83 | 29 – 39 |  |
|  | 5 | 34 | 3.6 | 0.640 | 2.14 | 28 – 40 |  |
|  | 6 | 35 | 3.9 | 0.688 | 2.15 | 29 – 41 |  |
|  | Overall | 33 | 3.2 | 0.728 | 1.66 | 29 – 38 |  |
| Affect | Min 0 | 2.56 | 1.2 | 0.830 | 0.51 | 0.85 – 3.78 | *-* |
|  | Min 5 | 2.31 | 1.3 | 0.831 | 0.53 | 0.91 – 3.43 |  |
|  | Min 10 | 2.17 | 1.1 | 0.826 | 0.45 | 0.98 – 3.27 |  |
|  | Min 15 | 2.13 | 0.9 | 0.777 | 0.41 | 0.48 – 3.22 |  |
|  | Min 20 | 1.85 | 1.1 | 0.786 | 0.49 | -0.47 – 3.35 |  |
|  | Min 25 | 1.44 | 1.1 | 0.597 | 0.69 | -0.47 – 3.30 |  |
|  | Min 30 | 1.42 | 1.2 | 0.686 | 0.68 | -0.99 – 3.49 |  |
|  | Overall | 1.25 | 1.1 | 0.488 | 0.81 | 0.47 – 3.12 |  |
| Self-efficacy | Min 0 | 7.58 | 1.6 | 0.904 | 0.49 | 5.87 – 9.01 | *-* |
|  | Min 5 | 7.44 | 1.7 | 0.883 | 0.57 | 6.02 – 9.52 |  |
|  | Min 10 | 7.77 | 1.5 | 0.812 | 0.63 | 6.23 – 9.52 |  |
|  | Min 15 | 7.88 | 1.2 | 0.765 | 0.59 | 6.19 – 9.68 |  |
|  | Min 20 | 7.94 | 1.1 | 0.654 | 0.63 | 5.97 – 9.49 |  |
|  | Min 25 | 7.73 | 1.1 | 0.636 | 0.63 | 6.63 – 9.79 |  |
|  | Min 30 | 8.21 | 0.8 | 0.505 | 0.57 | 7.52 – 9.90 |  |
|  | Overall | 8.71 | 1.2 | 0.862 | 0.43 | 6.71 – 9.20 |  |

| Supplementary Table 2. Group mean RPE_+15%GET_ inter- and intra-individual results for each time zone and overall. | | | | | | | |
| --- | --- | --- | --- | --- | --- | --- | --- |
| Variable | TZ | Mean | SD | ICC (2,1) | SEM | 95% CI | *CoV* |
| *V̇*_E_ | 1 | 87.1 | 11.4 | 0.827 | 4.76 | 73.9 – 100.3 | *2.8* |
|  | 2 | 95.9 | 18.7 | 0.933 | 4.84 | 82.5 – 109.3 |  |
|  | 3 | 94.0 | 17.4 | 0.944 | 4.13 | 82.5 – 105.4 |  |
|  | 4 | 94.0 | 18.2 | 0.951 | 4.03 | 82.8 – 105.1 |  |
|  | 5 | 94.6 | 19.2 | 0.950 | 4.29 | 82.7 – 106.5 |  |
|  | 6 | 94.3 | 20.8 | 0.936 | 5.27 | 79.7 – 108.9 |  |
|  | Overall | 93.3 | 16.9 | 0.963 | 3.26 | 84.3 – 102.3 |  |
| BF | 1 | 35 | 3.9 | 0.889 | 1.31 | 32 – 39 | *2.6* |
|  | 2 | 39 | 5.4 | 0.903 | 1.68 | 34 – 44 |  |
|  | 3 | 40 | 5.5 | 0.952 | 1.21 | 37 – 43 |  |
|  | 4 | 41 | 5.9 | 0.907 | 1.79 | 36 – 46 |  |
|  | 5 | 42 | 6.4 | 0.916 | 1.85 | 37 – 47 |  |
|  | 6 | 43 | 7.1 | 0.961 | 1.40 | 39 – 46 |  |
|  | Overall | 40 | 5.5 | 0.969 | 0.96 | 37 – 43 |  |
| Affect | Min 0 | 2.31 | 1.7 | 0.889 | 0.57 | -1.03 – 4.15 | *-* |
|  | Min 5 | 1.56 | 1.8 | 0.720 | 0.93 | -1.56 – 3.02 |  |
|  | Min 10 | 0.73 | 1.6 | 0.720 | 0.83 | -2.41 – 2.87 |  |
|  | Min 15 | 0.23 | 1.5 | 0.621 | 0.95 | -2.41 – 2.20 |  |
|  | Min 20 | -0.10 | 1.5 | 0.707 | 0.83 | -3.20 – 2.08 |  |
|  | Min 25 | -0.56 | 1.4 | 0.552 | 0.95 | -3.38 – 1.00 |  |
|  | Min 30 | -1.19 | 1.2 | 0.592 | 0.79 | -3.63 – 0.80 |  |
|  | Overall | -1.42 | 1.5 | 0.708 | 0.80 | -1.92 – 1.70 |  |
| Self-efficacy | Min 0 | 6.56 | 2.0 | 0.829 | 0.82 | 3.96 – 8.13 | *-* |
|  | Min 5 | 6.04 | 1.9 | 0.850 | 0.75 | 3.85 – 8.15 |  |
|  | Min 10 | 6.00 | 1.8 | 0.815 | 0.77 | 3.25 – 8.71 |  |
|  | Min 15 | 5.98 | 1.6 | 0.607 | 0.99 | 3.63 – 8.91 |  |
|  | Min 20 | 6.27 | 1.3 | 0.482 | 0.95 | 3.45 – 8.93 |  |
|  | Min 25 | 6.19 | 1.3 | 0.427 | 0.99 | 4.45 – 9.10 |  |
|  | Min 30 | 6.77 | 1.2 | 0.524 | 0.84 | 4.94 – 10.35 |  |
|  | Overall | 7.65 | 1.3 | 0.442 | 0.98 | 4.62 – 8.20 |  |
